# ANNUALIZED AND CUMULATIVE MEASURES OF ANTICHOLINERGIC EXPOSURE FOR RESEARCH AND CLINICAL APPLICATIONS

**DOI:** 10.1093/geroni/igac059.2312

**Published:** 2022-12-20

**Authors:** Hussein Khalil, Eva Stachler, Khalid Alamer, Jared Ludlow, Noll Campbell

**Affiliations:** Purdue University, West Lafayette, Indiana, United States; Purdue University, West Lafayette, Indiana, United States; Purdue University, West Lafayette, Indiana, United States; Indiana University Purdue University Indianapolis, Indianapolis, Indiana, United States; Purdue University, Indianapolis, Indiana, United States

## Abstract

Medications with anticholinergic properties are commonly used by older adults despite being associated with dementia.The anticholinergic total standardized daily dose (TSDD) is a continuous measure of exposure that has been associated with an increased risk of dementia at values >1095 over ten years in epidemiologic studies. We sought to determine a cumulative (cTSDD) and annualized (aTSDD) in a sample of community-dwelling older adults enrolled in the ongoing Reducing Risk of Dementia Through Deprescribing (R2D2) trial (NCT04270474). Participants were 65 years or older without dementia, attended at least one primary care visit within 12 months prior to enrollment, and were current users of strong anticholinergics according to the 2012 Anticholinergic Cognitive Burden Scale. Prescribed and over-the-counter medication details were collected during the baseline visit through self-report and included strength, frequency, units/dose and duration. The aTSDD was calculated for each participant assuming continuous use patterns throughout the year. The cTSDD was calculated by summing the aTSDD across the number of years since initiation. Of 66 participants, the median cTSDD was 2425 (IQR 5131), and 48 (72%) exceeded the threshold of dementia risk (>1095). Additionally, the aTSDD had a median of 730 (IQR 547), with 60 (90%) exceeding the threshold of dementia risk (109.5, one-tenth of the ten-year risk) while 11 (17%) exceeded dementia risk threshold of 1095 considering 1 year of exposure. Both measures identified the majority of anticholinergic users exceeding dementia risk thresholds despite some disagreement between the two approaches. However, both methods have potential for research and clinical applications.

